# Whole genome sequencing of “*Faecalibaculum rodentium*” ALO17, isolated from C57BL/6J laboratory mouse feces

**DOI:** 10.1186/s13099-016-0087-3

**Published:** 2016-02-12

**Authors:** Sooyeon Lim, Dong-Ho Chang, Sharon Ahn, Byoung-Chan Kim

**Affiliations:** Microbiomix and Immunity Research Center, Korea Research Institute of Bioscience and Biotechnology (KRIBB), 125 Gwahangno, Yuseong-gu, Daejeon, 34141 South Korea; Department of Biosystems and Bioengineering, University of Science and Technology (UST), 217 Gwahangno, Yuseong-gu, Daejeon, South Korea

**Keywords:** “*Faecalibaculum rodentium*” ALO17, Whole genome sequencing, Phylogeny, Comparative genomics

## Abstract

**Background:**

Intestinal microorganisms affect host physiology, including ageing. Given the difficulty in controlling for human studies of the gut microbiome, mouse models provide an alternative avenue to study such relationships. In this study, we report on the complete genome of “*Faecalibaculum rodentium*” ALO17, a bacterium that was isolated from the faeces of a 9-month-old female C57BL/6J mouse. This strain will be utilized in future in vivo studies detailing the relationships between the gut microbiome and ageing.

**Results:**

The whole genome sequence of “*F. rodentium*” ALO17 was obtained using single-molecule, real-time (SMRT) technique on a PacBio instrument. The assembled genome consisted of 2,542,486 base pairs of double-stranded DNA with a GC content of 54.0 % and no plasmids. The genome was predicted to contain 2794 open reading frames, 55 tRNA genes, and 38 rRNA genes. The 16S rRNA gene of ALO17 was 86.9 % similar to that of *Allobaculum stercoricanis* DSM 13633^T^, and the average overall nucleotide identity between strains ALO17 and DSM 13633^T^ was 66.8 %. After confirming the phylogenetic relationship between “*F. rodentium*” ALO17 and *A. stercoricanis* DSM 13633^T^, their whole genome sequences were compared, revealing that “*F. rodentium*” ALO17 contains more fermentation-related genes than *A. stercoricanis* DSM 13633^T^. Furthermore, “*F. rodentium*” ALO17 produces higher levels of lactic acid than *A. stercoricanis* DSM 13633^T^ as determined by high-performance liquid chromatography.

**Conclusion:**

The availability of the “*F. rodentium*” ALO17 whole genome sequence will enhance studies concerning the gut microbiota and host physiology, especially when investigating the molecular relationships between gut microbiota and ageing.

## Background

Interactions between animals and their intestinal microorganisms play a crucial role in host physiology [1–3]. For example, perturbations of the intestinal microbiota have been associated with immunological, metabolic, and neurological diseases [4, 5]. The metabolic activities of intestinal microorganisms directly affect food digestion, absorption, and energy production [6, 7]. The composition of the intestinal microbiota is also related to ageing of the host [8–10]. The gut microbiota changes dramatically between early and late stages of life, with a shift from *Lactobacillus* and *Bifidobacterium* to *Bacteroidetes* and *Clostridia* genera. This shift suggests a change from lactate metabolism to increased short-chain fatty acid (SCFA) production and carbohydrate metabolism as ageing progresses [8, 10–12]. To date, multiple studies have investigated the human intestinal microbiome to understand the relationships between the gut microbiota and host ageing [6, 8, 9]. However, difficulties controlling experimental conditions, including diet, medications, and housing status in complex human systems has contributed to inconsistencies in results from such studies. Mouse studies of the relationships between the microbiome and host ageing have provided better-controlled systems with consistent results. Langile et al. [10] divided female C57BL/6J mice into three age groups based on murine frailty index (FI) scores and reported that *Erysipelotrichaceae* was one of the dominant bacterial families colonising the guts of middle-aged mice (589 days old). These data are consistent with our unpublished data from investigations of the microbial diversity in mice of different ages. We found the most abundant operational taxonomic units (OTU) of middle-aged mice (18–21 months old) were related to *Allobaculum* species [13] within the family *Erysipelotrichaceae*. Using fresh and anoxic mice faeces and anaerobic culture techniques [13, 14], we isolated a strain closely related (99 % 16S rRNA gene sequence similarity) to the most abundant OTUs from the feces of C57BL/6J mice, and designated it “*Faecalibaculum rodentium*” ALO17 [13]. However, the overall intestinal microbiota, including the dominant strains present in the guts of different aged mice, has not been investigated in precise detail. In this study, the whole genome sequence of “*F. rodentium*” ALO17 was generated using a PacBio instrument and compared in silico to the previously-reported genome sequence of *Allobaculum stercoricanis* DSM 13633^T^.

## Methods

### Strain information

A strictly anaerobic bacterium, ALO17, was previously isolated [13] from the faeces of a 9-month-old female C57BL/6J mouse fed a standard experimental diet (cat. No. 2018S; Harlan Laboratories). The mouse was purchased from DBL Co. Ltd, Korea and housed in the specific pathogen-free facility of Korea Advanced Institute of Science and Technology (KAIST). All animal experiments were performed in accordance with the guidelines and policies for rodent experimentation provided by the Institutional Animal Care and Use Committee (IACUC) of KAIST. This study protocol was approved by the IACUC of KAIST (IACUC-13-140) [13]. The isolate was Gram-stain positive, non-motile, non-spore forming small rod, oxidase and catalase negative. As previously described [13, 15], strictly anaerobic techniques were used for the preparation of the DSM 104 medium (http://www.dsmz.de/microorganisms/medium/pdf/DSMZ_Medium104.pdf) and the cultivation process (the gas atmosphere was 100 % N_2_). Strain ALO17 was optimally cultivated in the DSM 104 broth at 37 °C and at pH 7 for 3 days under strict anaerobic condition. On the basis of polyphasic taxonomic experiments, we have proposed that the isolate be assigned to the family *Erysipelothricaceae* with the novel genus and species name, “*Faecalibaculum rodentium*” [13].

### Genome sequencing, assembly and annotation

Genomic DNA was purified from 3L cultures of “*F. rodentium*” ALO17 as previously described [13, 14]. Extracted DNA samples were sequenced using Pacific Biosciences RS sequencing technology (Pacific Biosciences, Menlo Park, CA), yielding > 50X coverage. Each sample was prepared as a 10-kb insert library using C2 chemistry and sequenced on the PacBio RS II (Pacific Biosciences), according to the manufacturer’s instructions. De novo genome assembly was performed using the CLCbio CLC Genomics Workbench v7.0.4 and PacBio SMRT Analysis 2.2.0 [16]. Annotation was completed using a homology search against the Clusters of Orthologous Groups (COG) and SEED databases [17, 18], respectively. SEED viewer [19] was used for subsystem functional categorization of the predicted ORFs and for visualization [20]. Average nucleotide identity (ANI) values were determined using the BLAST algorithm [21].

### Comparative genomics

Comparative genomic analyses were performed using BLAST and a robust pair-wise sequence alignment algorithm. 16S rRNA gene sequences with pairwise similarities >85 % were obtained from the EZtaxon database (http://www.ezbiocloud.net/eztaxon) for nine different species and used for phylogenetic analyses in MEGA6 [22]. Among the nine strains, whole genome sequences for *Eubacterium dolichum* DSM3991^T^, *Faecalitalea cylindroides* ATCC 27803^T^, *Holdemanella biformis* DSM 3989^T^, and *Allobaculum stercoricanis* DSM 13633^T^ were acquired from the NCBI database (http://www.ncbi.nlm.nih.gov/genome/genomes) and their ANI values [21] to “*F. rodentium*” ALO17 were calculated. For the calculation of ANI values, the query sequence was randomly cut into fragments of 1020 nucleotides and each was blasted against the subject genome. Following this, a genome tree was constructed using R software, and the most closely related sequences were determined according to the ANI values using the unweighted pair group method. A phylogenetic tree for the five strains was also generated using MEGA6. Fermentation-related genes in the “*F. rodentium*” ALO17 genome were categorized into functional groups using the annotated genome of *A. stercoricanis* DSM 13633^T^.

### Measurements of lactic acid concentration

The concentration of lactic acid in the anaerobic DSM 104 broth after 3 days cultivation at 37 °C were determined using an HPLC (1200 Series, Agilent Technology, USA) equipped with an Aminex 87H column (dimensions: 300*7.8, Bio-Rad, USA). The mobile phase was 0.01 M sulfuric acid with 0.5 ml/min flow rate at 40 °C. The average values with error ranges of lactic acid concentration were obtained from two different duplicate experiments.

### Quality assurance

Highly purified and intact genomic DNA was obtained from 3L cultures grown in the DSM 104 broth by the modified bead-beating technique [14, 23] and confirmed against the published genome obtained from the NCBI database. The 16S rRNA gene was extracted from the assembled contigs using the RAST annotation system. ANI values were converted into distances between the other genomes analysed.

## Results and discussion

**Fig. 1:**
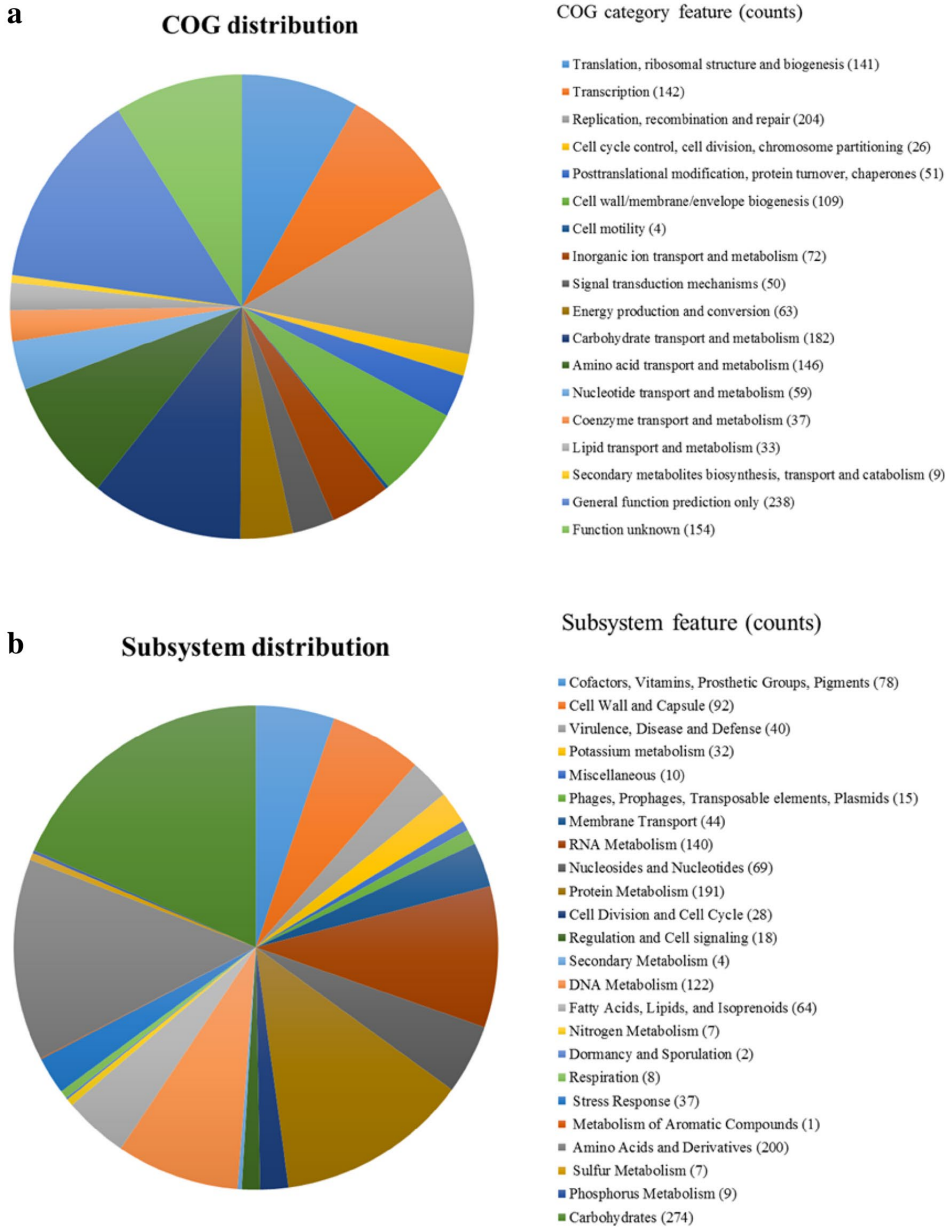
Statistics of annotated genes for “*F. rodentium*” Alo17 based on **a** COG and **b** SEED databases

**Fig. 2:**
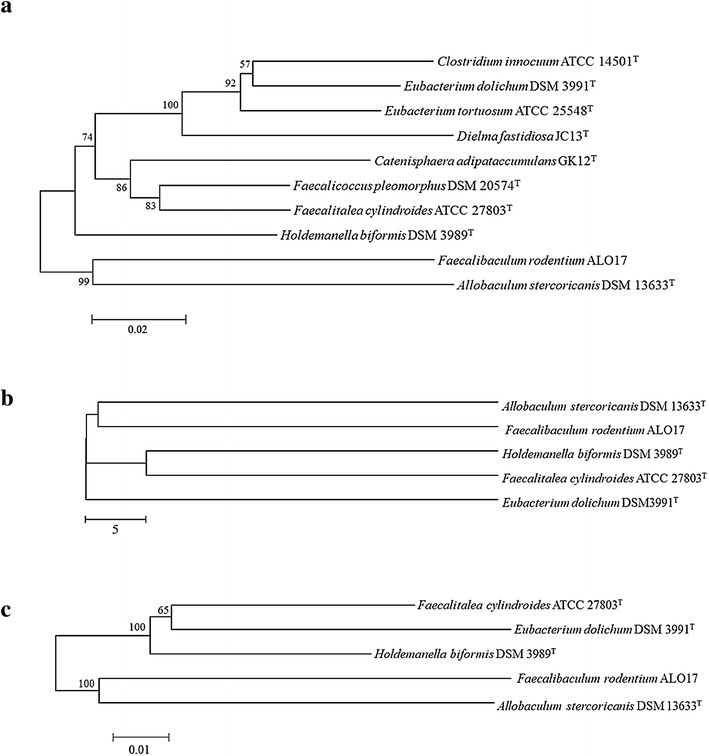
**a** Phylogenetic tree analysis of 10 strains using 16S rRNA sequence method (pairwise similarity >85 %). **b** Phylogenetic tree analysis of 5 strains using ANI (average nucleotide identity) methods. **c** Phylogenetic tree analysis of 5 strains using 16S rRNA sequence method. Bootstrap values (expressed as percentages of 1000 replication, >50 %) are shown at branching points. *Bar* 0.02 substitution per nucleotide position

The genome of “*F. rodentium*” ALO17 as assembled here consisted of a single circular DNA chromosome of 2,542,486 base pairs, a GC content of 54.0 %, and no plasmids. The genome contained 2583 predicted open reading frames (ORFs), 55 tRNAs, and 38 rRNAs. Analyses using SEED subsystem categorization and COG functional categorizations are shown in Fig. 1. SEED subsystem categorization predicted 1529 ORFs that encode known functional proteins, whereas 1054 ORFs were of unknown function. Among the ORFs with a predicted function, 274 were predicted to be for carbohydrate synthesis, 200 were predicted to be for amino acid synthesis, 191 for protein metabolism, 140 for RNA metabolism, 122 for DNA metabolism, and 78 for the production of cofactors, vitamins, prosthetic groups, and pigments. Additionally, 40 ORFs belonged to the fermentation category. There are five major roles for genes in this category. A total of 13 ORFs (32.5 %) were similar to genes responsible for the fermentation of acetyl-CoA to butyrate, 12 (30.0 %) were predicted to be involved in fermentations with mixed acids, 9 (22.5 %) in butanol biosynthesis, 4 (10.0 %) in lactate fermentation, and 2 (5.0 %) were predicted to be acetolactate synthase subunits. COG analyses assigned 1566 ORFs (91.0 % of all predicted ORFs) to functional categories. Among these, 815 ORFs (47.39 % of the COG-assigned ORFs) belonged to 5 primary categories: 204 ORFs belonged to Category L (replication, recombination, and repair), 182 to Category G (carbohydrate transport and metabolism), 146 to Category E (amino acid transport and metabolism), 142 to Category K (transcription), and 141 to Category J (translation, ribosomal structure and biogenesis). For comparative genomic analyses, two methods were used: ANI and 16S rRNA gene sequencing. Nine bacterial species with pairwise similarities >85 % for the 16S rRNA gene, compared to “*F. rodentium*” ALO17, were selected. Among those selected, whole genome sequences for four species were in the NCBI database. A phylogenetic tree was constructed based on the 16S rRNA gene sequences. ANI values calculated between ALO17, *E. dolichum* DSM3991^T^, *F. cylindroides* ATCC 27803^T^, *H. biformis* DSM 3989^T^, *A. stercoricanis* DSM 13633^T^, were 63.9, 65.8, 66.2 and 66.8 %, respectively. Strain ALO17 clustered with strain DSM 13633^T^ in the phylogenetic tree, which was supported by a high bootstrap value and ANI dendrogram (Fig. 2). Due to their high level of relatedness, additional detailed genomic analyses were performed between “*F. rodentium*” ALO17 and other 4 reference of *A. stercoricanis* DSM 13633^T^, *H. biformis* DSM 3989^T^, *F. cylindroides* ATCC 27803^T^ and *E. dolichum* DSM3991^T^. This comparison of the fermentation related genes is summarised in Table 1. “*F. rodentium*” ALO17 had a single homologue predicted to encode a protein that stimulates sugar/maltose fermentation. Additionally, subsystems for acetolactate synthase subunits, the fermentation of acetyl-CoA to butyrate, butanol biosynthesis, lactate fermentation, and mixed acid fermentation were present in both “*F. rodentium*” ALO17 and *A. stercoricanis* DSM 13633^T^ genomes. The numbers of genes in “*F. rodentium*” ALO17 and *A. stercoricanis* DSM 13633^T^ predicted to be involved in fermentation subsystems were 40 and 23, respectively. “*F. rodentium*” ALO17 had more genes predicted to be involved in specific functions, compared to *A. stercoricanis* DSM 13633^T^. Such genes were predicted to encode for 3-hydroxybutyryl-CoA dehydrogenase (EC 1.1.1.157), 3-hydroxybutyryl-CoA dehydrogenase (EC 1.1.1.157), electron transfer flavoprotein (alpha and beta subunit), 3-hydroxybutyryl-CoA dehydrogenase (EC 1.1.1.157), pyruvate formate-lyase (EC 2.3.1.54), L-lactate dehydrogenase (EC 1.1.1.27), pyruvate formate-lyase (EC 2.3.1.54), and pyruvate formate-lyase activating enzyme (EC1.97.1.4). These data suggest that this isolate has stronger fermentation activity than *A. stercoricanis* DSM 13633^T^. To test this hypothesis, the lactic acid concentrations were measured in the growth medium of “*F. rodentium*” ALO17 and *A. stercoricanis* DSM 13633^T^ by high-performance liquid chromatography. After three days of incubation at 37 °C, the lactic acid concentrations were observed to be 9.5 ± 0.6 mM (strain ALO17) and 4.9 ± 0.8 mM (*A. stercoricanis* DSM 13633^T^), respectively. Previous study of *A. stercoricanis* DSM 13633^T^ showed the level of lactic acid was 3.5 mM [24]. In summary, despite their phylogenetic clustering, “*F. rodentium*” ALO17 differed from *A. stercoricanis* DSM 13633^T^ in lactic acid production. We hypothesize that “*F. rodentium*” ALO17 is an obligate anaerobe that replaces *Lactobacilli* and *Bifidobacterium* in middle-aged mice. *Lactobacilli* and *Bifidobacterium* are primary lactic acid producers in young mice; however, as the gut becomes strictly anaerobic with age, “*F. rodentium*” may become dominant. Thus, dominance of lactate producers and lactate production in the animal gut might be inversely related to ageing [10].

**Table 1:** Comparison of fermentation related organism between “*F. rodentium*” ALO17 and other 4 references of *A. stercoricanis* DSM 13633^T^, *H. biformis* DSM 3989^T^, *F. cylindroides* ATCC 27803^T^ and *E. dolichum* DSM3991^T^

Subsystem	Role description	Number of genes				
		Alo17	13633^T^	3989^T^	27803^T^	3991^T^
Butanol biosynthesis	Alcohol dehydrogenase (EC 1.1.1.1)	1	1	1	1	1
Butanol biosynthesis	NADH-dependent butanol dehydrogenase A (EC 1.1.1.-)	1	1	1	0	1
Butanol biosynthesis	Pyruvate formate-lyase (EC 2.3.1.54)	3	1	5	4	1
Butanol biosynthesis	3-Hydroxybutyryl-CoA dehydrogenase (EC 1.1.1.157)	3	1	1	1	1
Butanol biosynthesis	Acetyl-CoA acetyltransferase (EC 2.3.1.9)	1	1	2	2	1
Fermentations: mixed acid	Alcohol dehydrogenase (EC 1.1.1.1)	1	1	1	1	1
Fermentations: mixed acid	l-lactate dehydrogenase (EC 1.1.1.27)	2	1	2	2	1
Fermentations: mixed acid	Sugar/maltose fermentation stimulation protein homolog	1	0	0	0	0
Fermentations: mixed acid	Pyruvate formate-lyase (EC 2.3.1.54)	3	1	5	4	1
Fermentations: mixed acid	Pyruvate formate-lyase activating enzyme (EC 1.97.1.4)	3	1	4	4	1
Fermentations: mixed acid	Phosphate acetyltransferase (EC 2.3.1.8)	1	1	1	0	1
Fermentations: Mixed acid	Acetate kinase (EC 2.7.2.1)	1	1	1	1	1
Acetolactate synthase subunits	Acetolactate synthase large subunit (EC 2.2.1.6)	1	1	0	0	0
Acetolactate synthase subunits	Acetolactate synthase small subunit (EC 2.2.1.6)	1	1	0	0	0
Fermentations: lactate	l-Lactate dehydrogenase (EC 1.1.1.27)	2	1	2	2	1
Fermentations: lactate	Phosphate acetyltransferase (EC 2.3.1.8)	1	1	1	0	1
Fermentations: lactate	Acetate kinase (EC 2.7.2.1)	1	1	1	1	1
Acetyl-CoA fermentation to butyrate	3-Hydroxybutyryl-CoA dehydratase (EC 4.2.1.55)	1	1	1	1	0
Acetyl-CoA fermentation to butyrate	Electron transfer flavoprotein, beta subunit	2	1	1	1	0
Acetyl-CoA fermentation to butyrate	Electron transfer flavoprotein, alpha subunit	2	1	1	1	0
Acetyl-CoA fermentation to butyrate	3-Hydroxybutyryl-CoA dehydrogenase (EC 1.1.1.157)	3	1	1	1	0
Acetyl-CoA fermentation to butyrate	3-Hydroxyacyl-CoA dehydrogenase (EC 1.1.1.35)	3	1	1	1	0
Acetyl-CoA fermentation to butyrate	Acetyl-CoA:acetoacetyl-CoA transferase, alpha subunit (EC 2.8.3.8)	1	1	2	1	0
Acetyl-CoA fermentation to butyrate	Acetyl-CoA Acetyltransferase (EC 2.3.1.9)	1	1	2	2	0

### Initial findings

The genome of “*F. rodentium*” ALO17 contained 2583 predicted open reading frames (ORFs), 55 tRNAs, and 38 rRNAs. Among them, 40 and 23 genes in “*F. rodentium*” ALO17 and *A. stercoricanis* DSM 13633^T^ predicted to be involved in fermentation subsystems. This result suggest that “*F. rodentium*” ALO17 has more fermentation activity than *A. stercoricanis* DSM 13633^T^. The lactic acid concentrations were measured by high-performance liquid chromatography and “*F. rodentium*” ALO17 produces higher levels of lactic acid than *A. stercoricanis* DSM 13633^T^.

### Future directions

This is the first report on the complete genome sequence of “*F. rodentium*” ALO17. This bacterium was isolated from a 9-month-old laboratory mouse and its genome was sequenced using PacBio SMRT technology. “*F. rodentium*” ALO17 is phylogenetically related to *A. stercoricanis* DSM 13633^T^, which belongs to the family *Erysipelotrichaceae*, and this family of bacterium is dominant in the gut of middle-aged mice [10]. Considering the robust production of lactic acid in this isolate, further analyses will provide useful information regarding the relationships between gut microbiota, lactate metabolism, and host ageing.

## Availability of supporting data

The genome sequence of “*F. rodentium*” ALO17 was deposited in the Genbank under the accession number of CP011391.
